# Screening for Chronic Kidney Disease: Preventing Harm or Harming the Healthy?

**DOI:** 10.1371/journal.pmed.1001345

**Published:** 2012-11-20

**Authors:** Maarten W. Taal

**Affiliations:** Department of Renal Medicine, Royal Derby Hospital, Uttoxeter Road, Derby, Derbyshire, United Kingdom

## Abstract

Maarten Taal discusses the potential benefits, risks, and evidence base relating to screening policies for chronic kidney disease.

**Linked Research Article**

This Perspective discusses the following new study published in *PLOS Medicine*:

Echouffo-Tcheugui JB, Kengne AP (2012) Risk Models to Predict Chronic Kidney Disease and Its Progression: A Systematic Review. PLoS Med 9(11): e1001344. doi:10.1371/journal.pmed.1001344

A systematic review of risk prediction models conducted by Justin Echouffo-Tcheugui and Andre Kengne examines the evidence base for prediction of chronic kidney disease risk and its progression, and suitability of such models for clinical use.

Advances in understanding the pathogenesis and natural history of diseases as well as developments in medical technology have made it possible to diagnose a large number of diseases at early stages, often in asymptomatic individuals. It is intuitive to believe that earlier diagnosis is beneficial because it creates the opportunity to intervene and prevent progression. This simple paradigm may hold true for some conditions, e.g., hypertension, but, it is increasingly clear that for other diseases early diagnosis may not necessarily be beneficial—e.g., in prostate cancer, since not all cases identified will progress to cause symptoms or premature death. Thus there are calls for health professionals to critically appraise the evidence relating to screening policies, to prevent overdiagnosis (“harming the healthy”) [1]. A new systematic review in this week's *PLOS Medicine* by Justin Echouffo-Tcheugui and Andre Kengne, which examined the evidence base for prediction of chronic kidney disease (CKD) risk and its progression, offers a chance to consider these questions for the clinical management of CKD [2].

CKD initially seems to be a disease for which screening to facilitate early diagnosis would be beneficial. CKD is relatively common [3], is asymptomatic until advanced stages, progresses over several years, and leads to end-stage kidney disease (ESKD) as well as several other adverse outcomes [4]–[7]. Nevertheless, while evidence indicates benefit associated with CKD screening in people with diabetes or hypertension, we are still some way from understanding the best strategy for CKD screening in asymptomatic people without such conditions. In short, will general population screening for CKD be effective in identifying the “truly ill” or simply “harm the healthy”?

## Accuracy of Screening Tests

Diagnosis of CKD requires evidence of kidney damage and/or reduced glomerular filtration rate (GFR) that is sustained over at least 3 months [8]. The tests generally used to detect CKD are estimated GFR (eGFR) derived from serum creatinine concentration and urinary albumin to creatinine ratio (ACR), a measure of albuminuria. Unfortunately, these tests have significant limitations. Firstly, the formula most widely used for eGFR (the MDRD equation) systematically underestimates GFR above the threshold below which CKD may be diagnosed without additional evidence of kidney damage [9]. This equation is also not well validated in the elderly, leading some nephrologists to question the validity of diagnosing CKD based on eGFR alone [10]. A more accurate equation, CKD-EPI, that performs better at higher GFR values has been developed and may replace the MDRD equation, but performance in the elderly is also uncertain [11]. Recently, a new equation that estimates GFR from serum creatinine and cystatin C has been shown to correctly reclassify some patients as not having CKD, thus reducing overdiagnosis [12]. Urinary ACR correlates closely with urinary albumin excretion, but mild albuminuria may be provoked by fever or exercise, and longitudinal studies have shown that microalbuminuria may regress in people with diabetic [13] and non-diabetic CKD [3]. Despite these limitations, studies utilising MDRD eGFR and urinary ACR have shown that these admittedly imperfect measures do serve as predictors of risk by identifying eGFR and albuminuria as strong independent risk factors for increased mortality, cardiovascular events, acute kidney injury [4]–[6], and venous thromboembolism [7].

## Potential Benefits of Screening for CKD

Early diagnosis of CKD creates the opportunity for intervention to improve prognosis. Whereas there is clear evidence that even minor reductions in GFR and mild albuminuria are independent risk factors for adverse outcomes, evidence that intervention alters the prognosis in people with mild forms of CKD is sparse. Treatment with inhibitors of the renin-angiotensin-aldosterone system (RAASi) has been shown to slow progression of CKD in patients with diabetes [14] or proteinuria [15], but evidence of benefit in others with CKD is limited. Similarly, clear evidence that RAASi treatment lowers the cardiovascular risk associated with CKD is limited to those with diabetes [16] or is indirect [17]. Lipid lowering therapy has recently been shown to reduce the risk of atherosclerotic events in people with CKD stages 3–5, recruited from secondary care [18], but whether these benefits would be achieved in those with milder forms of CKD is untested.

## Potential Harm from Screening for CKD

Potential harms resulting from screening for CKD in the general population include the psychological effects of receiving a diagnosis of CKD as well as the burden of potentially having to undergo additional investigation or referral to secondary care. In addition, a CKD diagnosis may harm a person's potential for employment and obtaining life insurance. For health care systems, the risks of screening for CKD include the costs of increased patient visits and tests as well as opportunity costs due to the fact that resources are not available for other services. As far as I am aware, there are no published randomised trials of screening for CKD and the potential harms have not been studied.

## Conclusion

Despite expectations that screening the general population without diabetes or hypertension for CKD would afford net benefit, there is insufficient evidence to date to inform a recommendation. The United States Preventative Services Task Force (USPSTF) has recently confirmed this view after a comprehensive review of the evidence [19]. Efforts to develop risk prediction tools to target screening towards those at higher risk are likely to improve the efficiency of screening programmes, but as noted by Echouffo-Tcheugui and Kegne, published risk predicition formulae require further development and external validation [2]. In the absence of evidence showing benefit from population screening for CKD, most guidelines recommend that testing should be directed to people with known risk factors [8],[20], but in light of improved diagnostic tests and novel risk prediciton tools, further research is required to establish the most cost-effective approach.

## Abbreviations

ACR: urinary albumin to creatinine ratio
CKD: chronic kidney disease
eGFR: estimated glomerular filtration rate
GFR glomerular filtration rate
RAASi: renin-angiotensin-aldosterone system inhibitors
